# Chrysin Inhibits High Glucose-Induced Migration on Chorioretinal Endothelial Cells via VEGF and VEGFR Down-Regulation

**DOI:** 10.3390/ijms21155541

**Published:** 2020-08-02

**Authors:** Zhen-Yu Liao, I-Chia Liang, Hsin-Ju Li, Chia-Chun Wu, Huey-Ming Lo, Der-Chen Chang, Chi-Feng Hung

**Affiliations:** 1 Department of Internal Medicine, Shin Kong Wu Ho-Su Memorial Hospital, Taipei 111, Taiwan; ariex327@mail2000.com.tw; 2 Department of Ophthalmology, Tri-Service General Hospital, National Defense Medical Center, Taipei 11490, Taiwan; ysonyaliang@gmail.com; 3 Ph.D. Program in Nutrition and Food Science, Fu Jen Catholic University, New Taipei City 24205, Taiwan; 4 School of Medicine, Fu Jen Catholic University, New Taipei City 24205, Taiwan; sakumanatsumi@gmail.com; 5 Graduate Institute of Biomedical and Pharmaceutical Science, Fu Jen Catholic University, New Taipei City 24205, Taiwan; bobwu80113@gmail.com; 6 Division of Cardiology, Fu Jen Catholic University Hospital, New Taipei City 24205, Taiwan; m006459@ms.skh.org.tw; 7 Department of Mathematics and Statistics and Department of Computer Science, Georgetown University, Washington, DC 20057, USA; Chang@georgetown.edu; 8 MS Program Transdisciplinary Long Term Care, Fu Jen Catholic University, New Taipei City 24205, Taiwan; 9 Ph.D. Program in Pharmaceutical Biotechnology, Fu Jen Catholic University, New Taipei City 24205, Taiwan

**Keywords:** high glucose, chrysin, chorioretinal endothelial cell, vascular endothelial growth factor (VEGF)

## Abstract

Background: Diabetes mellitus (DM) is a chronic inflammatory disease, which causes multiple complications. Diabetic retinopathy (DR) is among these complications and is a dominant cause of vision loss for diabetic patients. Numerous studies have shown that chrysin, a flavonoid, has many biological activities such as anti-oxidation and anti-inflammation. However, it is rarely used in ocular diseases. In this study, we examined the inhibitory effects of flavonoid on high glucose induced migration of chorioretinal endothelial cells (RF/6A cells) and its mechanism. Materials and methods: The viability of RF/6A cells treated with chrysin was examined with a 3-(4,5-dimethyl-2-thiazolyl)-2,5-diphenyl-2H-tetrazolium bromide (MTT) assay. The migration of RF/6A cells was assessed by the transwell migration and scratch wound assays. The expression of AKT, ERK, vascular endothelial growth factor (VEGF), HIF−1α and MMP-2 were determined by western blotting. To observe the mRNA expression of VEGF receptor (VEGFR), qRT-PCR, was utilized. Results: The results showed that chrysin can dose-dependently inhibit the RF/6A cell migration in vitro transwell and the scratch wound assays which are induced by high glucose. After pretreatment of RF/6A cells with different concentrations of chrysin, they did not produce any cytotoxicity in MTT assay. Moreover, chrysin down-regulated both phosphorylated AKT and ERK, as well as attenuated the expression levels of MMP-2. It also decreased the expression of the VEGF transcription factor and VEGF. Furthermore, it was shown that chrysin could suppress the protein and mRNA expression levels of VEGFR. Conclusion: The results indicate that chrysin could down-regulate the phosphorylation of AKT, ERK and MMP-2 and reduce the effects of VEGF and VEGFR in a high glucose environment. It further inhibits the high glucose-induced migration of RE/6A cells. Therefore, chrysin may have the potential for visual protection.

## 1. Introduction

Diabetes mellitus (DM) is a type of metabolic disorder related to inflammation. It can cause both macro- and microvascular complications which include cardiovascular diseases, retinopathy, nephropathy, neuropathy, and poor wound healing. Diabetic retinopathy (DR) is a retinal microvascular disease characterized by inflammatory and angiogenic pathways. Clinical assessments of DR reveal typical microvascular features of initial retinal hemorrhages, lipid exudates, cotton wool spots, and finally, neovascularization. Changes at the retinal neurovascular unit, a term referring to the intricate functional coupling between neurons, glial cells, and blood vessels (the components of blood-retinal barrier), are susceptible to diabetes [1,2]. Progressive diabetes damage can lead to changes in the cellular environment. Vascular endothelial growth factor (VEGF) of glial cells increases and releases inflammatory cytokines, which leads to the destruction of the blood-retinal barrier as well as to angiogenesis, which is the main cause of DR vision damage [2]. The walls of the new vessels are not intact and therefore fluid exudation occurs, which results in vision impairment [3].

Angiogenesis was known to be a multi-factorial process, and cell migration was one of the initial processes. VEGF was known to play an important role in angiogenesis. The migration of chorioretinal endothelial cell (EC) was noted to be related to AKT and ERK phosphorylation [4,5]. Matrix metalloproteinase-2 (MMP-2) also participated in cell migration processes [6,7]. Additionally, a previous study revealed that chrysin, a kind of flavonoid, can decrease the expression of VEGF by inhibiting HIF-1α protein synthesis and decreasing its structure stability [8].

Flavonoids are the largest group of heterocyclic compounds in plants and exist in vegetables and fruits of any color, such as tomatoes, grapes, many kinds of nuts, beans, and even green tea. Currently, over 5000 naturally occurring flavonoids have been characterized. Common flavonoids include procyanidins, quercetin, chrysin, anthocyanin, apigenin, limonin, and catechin. Among them, quercetin’s structure has been known to modulate and strengthen the immune system [9]. Chrysin’s structure not only has the same immune modulating effect as quercetin’s structure, but has also been noted to have characteristics of anti-tumor and tumor cell apoptosis induction [10,11]. In our previous work, we noted that chrysin can protect keratinocyte from UVA and UVB damage, decrease reactive oxygen species (ROS) formation, and prevent cells from apoptosis. Further, animal experiments showed that chrysin can be absorbed by skin in mice without irritable injury [12]. We also found that chrysin may have therapeutic potential against inflammatory skin diseases [13]. Chrysin was also known to inhibit lipopolysaccharide (LPS) induced angiogenesis by down-regulating VEGF [14]. However, the anti-neovascular function of chrysin in diabetic retinopathy is not yet clear.

This study aimed to understand the role of chrysin in ophthalmology and investigate its role in high-glucose related chorioretinal EC migration. We attempt to understand if chrysin can protect chorioretinal EC from proliferation through the experimental model of high-glucose environment simulated diabetes. The problem of DM worsens by day, while drug resistance and side effects of the hypoglycemic drugs make the situation even worse and stricter [15]. Searching for effective ingredients, extracted from natural products, which can be used as alternative treatment or prevention is imperative, and chrysin is one of these discoveries. Our results will help evaluate whether chrysin has further potential in the prevention or treatment of diabetes and related complications.

## 2. Results

### 2.1. Chrysin Showed No Cytotoxicity to RF/6A Cells

**Figure 1:**
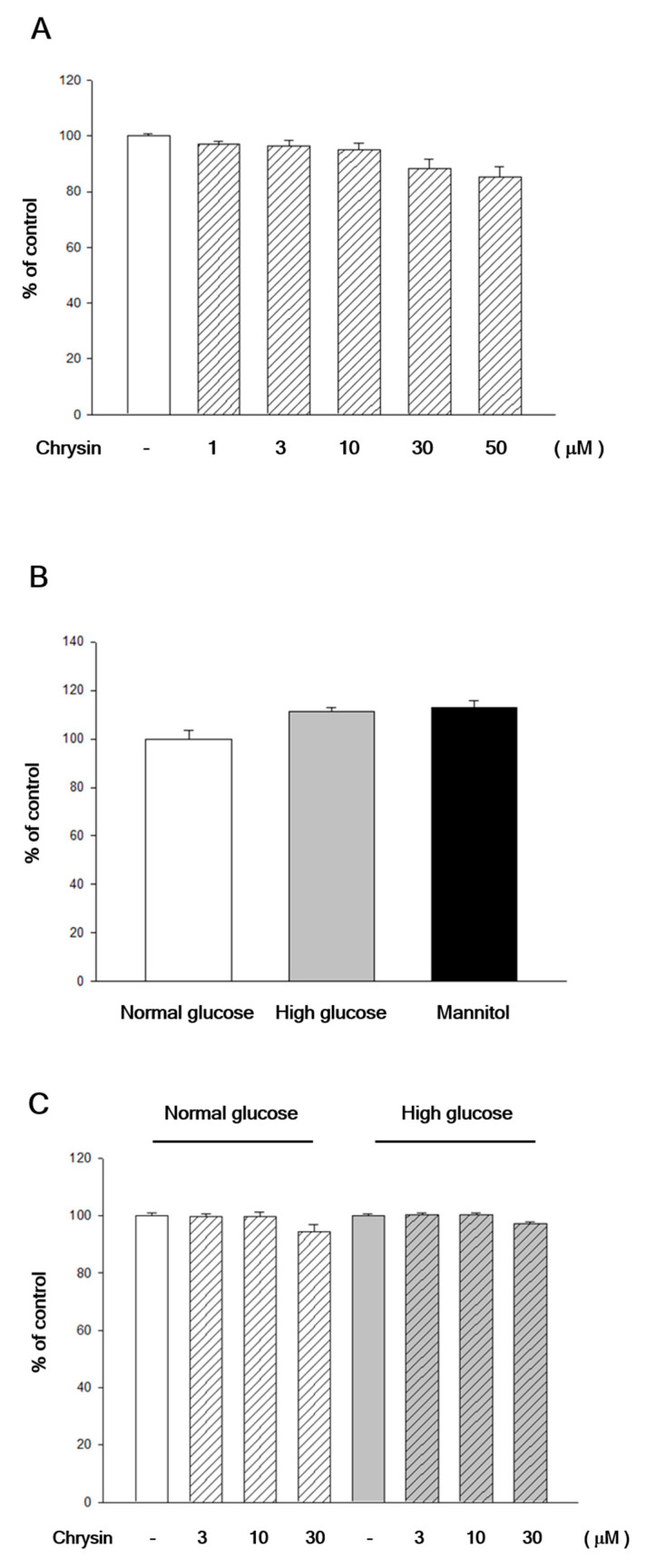
Cell viability was tested under different concentrations of chrysin or sugar. (**A**) The cell viability of RF/6A cells treated with chrysin at different concentrations. RF/6A cells treated with chrysin at different concentrations (1, 3, 10, 30, and 50 µM) and the 3-(4,5-dimethyl-2-thiazolyl)-2,5-diphenyl-2H-tetrazolium bromide (MTT) assay was then performed for cytotoxicity. (**B**) RF/6A cells were cultured in normal glucose (5.5 mM), high glucose (30 mM), or mannitol (30 mM) medium for 24 h and the MTT assay was then performed for cell viability. Results are expressed as the percentage of control and mean ± standard error (SE). (**C**) RF/6A cells were treated with chrysin at different concentrations (1, 3, 10, 30, and 50 µM) for 24 h and then cultured in normal glucose or high glucose, before the MTT assay was performed for viability. Results are expressed as the percentage of control and mean ± SE.

To confirm that the decrease of high-glucose induced migration was not due to the toxicity of chrysin, an MTT assay was used. Adding different concentrations of chrysin showed no cytotoxic effect on cell survival (Figure 1A), even under a very high concentration of 30 µM or 50 µM. The different osmolarity of high-glucose and normal-glucose culture mediums also showed no influence to cell survival compared with a control group of mannitol (30 mM) simulated hyperosmotic status (Figure 1B). Further experiments also showed that under different osmolarity of high glucose and normal glucose medium, different concentrations of chrysin had no effect on cell survival (Figure 1C).

### 2.2. Chrysin Inhibited High-Glucose Induced RF/6A Migration via Inhibiting AKT and ERK Phosphorylation and Decreasing MMP-2 Expression

**Figure 2:**
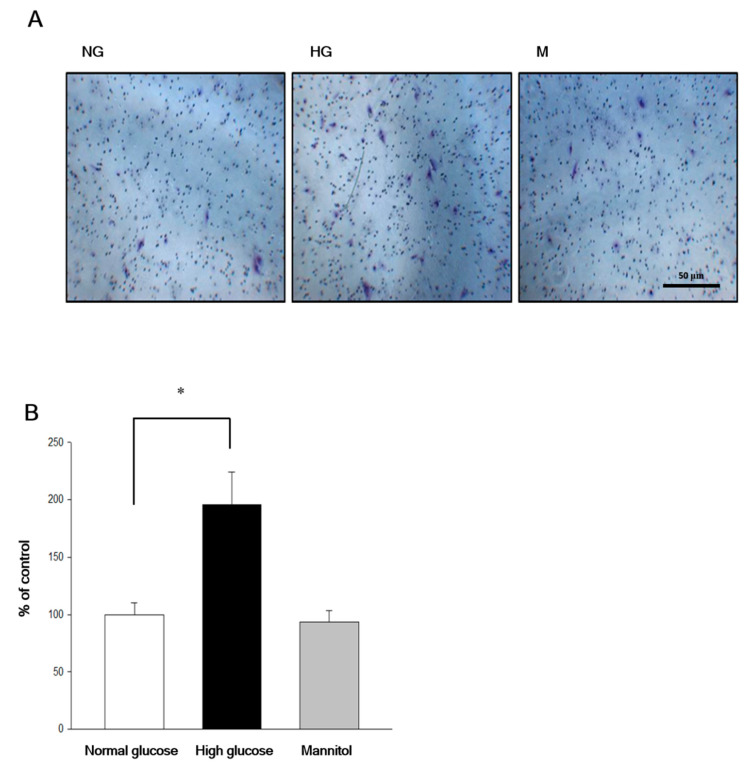
Chrysin inhibited high-Glucose induced RF/6A migration by the transwell assay. (**A**) RF/6A cells’ migration was promoted by high glucose. RF/6A cells were cultured in normal glucose, high glucose, or mannitol medium for 24 h. The transwell assay was performed for cell migration afterwards. NG: normal glucose; HG: high glucose; M: mannitol. (**B**) The quantitative results of the transwell cell migration assay for RF/6A cells cultured in normal glucose (5.5 mM), high glucose (30 mM), or mannitol (30 mM) medium for 24 h. Results are expressed as a percentage of control and mean ± SE. *p < 0.05, vs. control. Scale bar, 50 μm.

Transwell experiments were used to understand the effect of chrysin on high-glucose-induced RF/6A migration. RF/6A migration could be induced only under high-glucose, but not the normal-glucose environment. However, the hyperosmotic state medium does not affect cell migration (Figure 2).

**Figure 3:**
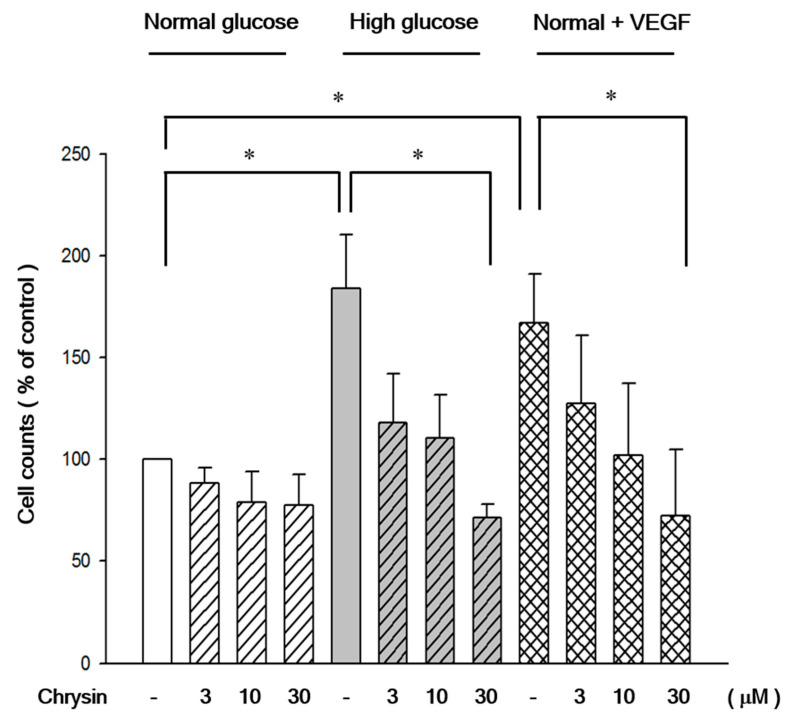
The quantitative results of the transwell assay for RF/6A cells treated chrysin and cultured in normal glucose, high glucose, or normal glucose with VEGF. The inhibitory effects of RF/6A cell migration treated with chrysin at different concentrations (3, 10, 30 µM) for 24 h and then cultured in normal glucose (5.5 mM), high glucose (30 mM), or normal glucose with vascular endothelial growth factor (VEGF) (25 pg/mL), for 24 h. The transwell assay was performed subsequently for cell migration. Results are expressed as a percentage of control and mean ± SE. *p < 0.05, vs. control, *p < 0.05, vs. high glucose group control, *p < 0.05, vs. normal with VEGF group control.

**Figure 4:**
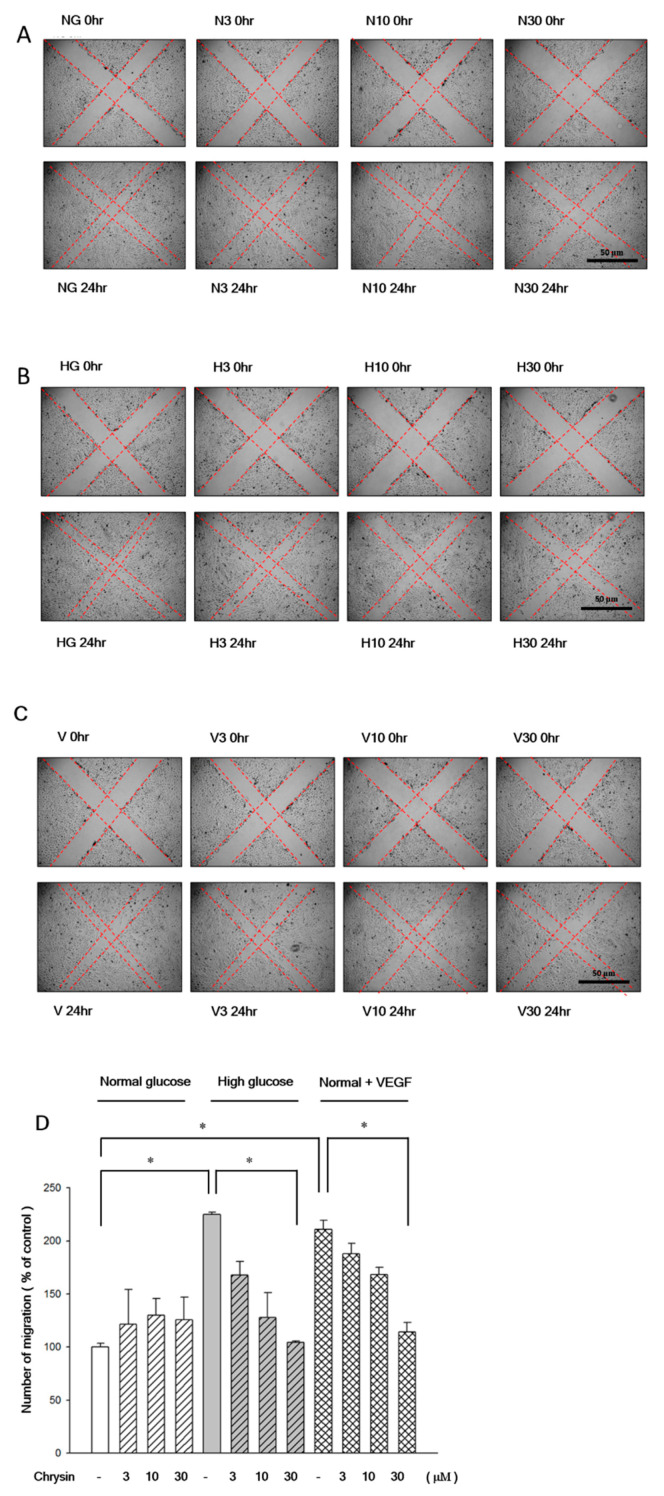
Chrysin inhibited high-glucose induced RF/6A migration by scratch wound assay. The inhibitory effects of RF/6A cell migration treated with chrysin in normal glucose (5.5 mM), high glucose (30 mM), or normal glucose with 25 pg/mL VEGF through the wound healing assay. RF/6A cells were treated chrysin at different concentrations (3, 10, 30 µM) for 24 h and then cultured in normal glucose (**A**) high glucose (**B**) or normal glucose with VEGF (**C**) for 24 h. The wound-healing assay was performed for cell migration. (**D**) The quantitative results of the wound healing assay for RF/6A cells treated chrysin and cultured in normal glucose, high glucose, or normal glucose with VEGF. Results are expressed as a percentage of control and mean ± SE. *p < 0.05, vs. control, *p < 0.05, vs. high glucose group control, *p < 0.05, vs. normal with VEGF group control. Scale bar, 50 μm.

RF/6A cells pre-treated with chrysin (3 µM, 10 µM, and 30 µM) represented resistance to high-glucose induced cell migration in the transwell assay (Figure 3). VEGF was added to the normal-glucose group to confirm its effect on inducing RF/6A migration even under the normal-glucose environment in advance. Our data showed that the VEGF-induced migration could also be inhibited by the pretreatment of chrysin. These inhibitory effects occurred in a concentration dependent manner (Figure 3). Moreover, these effects were confirmed once more by the scratch wound assays. We also found that chrysin dose-dependently inhibits the migration of RF/6A in experiments of scratching wound (Figure 4).

**Figure 5:**
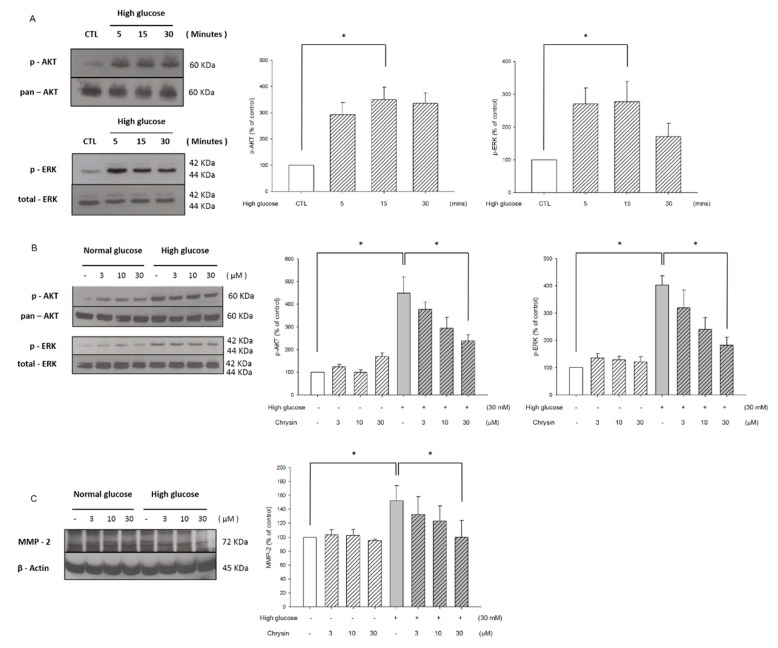
Chrysin inhibited AKT and ERK phosphorylation and decreasing MMP-2 expression. (**A**) The time course for high glucose up-regulated AKT and ERK phosphorylation. RF/6A cells were cultured in a high glucose medium (30 mM) and harvested at different time points (5, 15, 30 min). Subsequently, the western blot analysis was performed for the phosphorylation of AKT and ERK. The quantification data is shown on the right panel. (**B**) Chrysin down-regulated AKT and ERK phosphorylation induced by high glucose. RF/6A cells were treated with chrysin and then harvested after changing high glucose for 15 min. A western blot analysis was performed for the phosphorylation of AKT and ERK. The quantification data is shown on the right panel. (**C**) Chrysin suppressed MMP-2 expression levels induced by high glucose with the quantification data being shown on the right panel. All data was acquired by means of ± SE from at least three independent experiments. *p < 0.05, vs. control.

Next, we further studied the roles of AKT and ERK in the inhibition of high glucose-induced RF/6A migration by chrysin. Results showed that the highest expression of AKT and ERK phosphorylation were developed after 15 min of high-glucose stimulation (Figure 5A). After pretreatment of chrysin, the phosphorylation of AKT and ERK was found to decrease (Figure 5B). Moreover, results showed that chrysin has down-regulation of MMP-2 expression under the high-glucose environment in RF/6A cells (Figure 5C).

### 2.3. Chrysin Inhibits the HIF-1α and VEGF Expression

**Figure 6:**
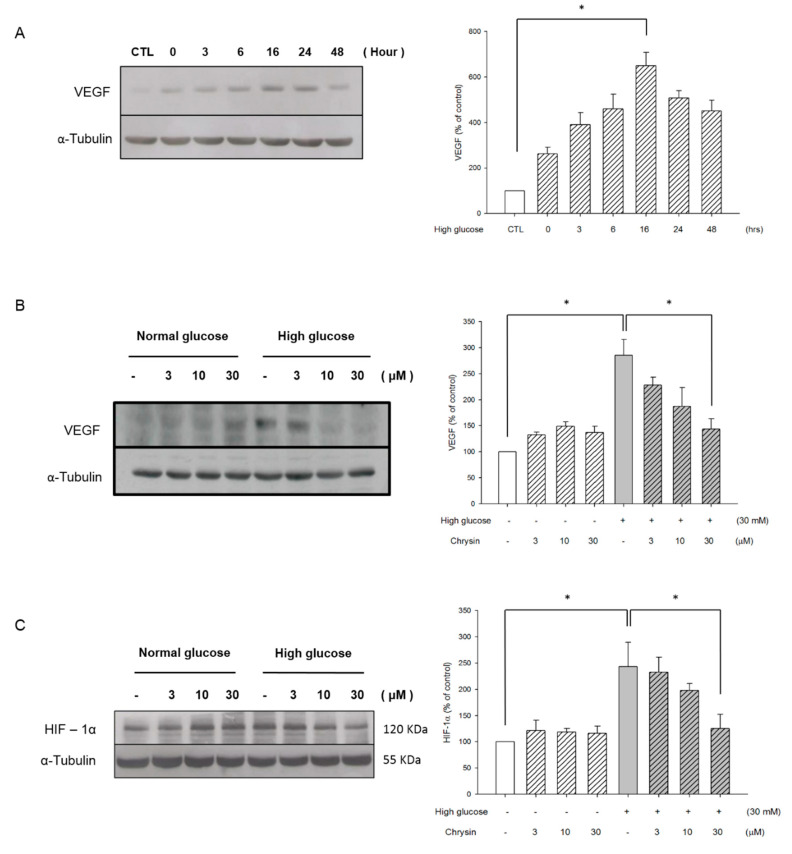
Chrysin Inhibited the HIF-1α and VEGF expression. (**A**) The time course for VEGF expression levels induced by high glucose. RF/6A cells were cultured in the high glucose medium (30 mM) and harvested at different time points (0, 3, 6, 16, 24, 48 h). Subsequently, a western blot analysis was performed for the expression levels of VEGF with the quantification data being displayed on the right panel. (**B**) The effects of the intracellular expression levels of VEGF with chrysin treatment and high glucose with the quantification data being shown on the right panel. (**C**) The effects of the expression levels of HIF-1α with chrysin treatment and high glucose with the quantification data being shown on the right panel. All data was acquired by means of ± SE from at least three independent experiments. *p < 0.05, vs. control.

The amount of VEGF expressed at different time points of high-glucose stimulation (0, 3, 6, 16, 24, 48 h) was confirmed. Highest VEGF expression was found at 16 h (Figure 6A). In the high-glucose environment, we found that the expression of intracellular VEGF decreased after pre-treatment of chrysin in RF/6A cells (Figure 6B). Furthermore, we studied the effect of chrysin on the VEGF transcription factor, HIF-1α, and found that high glucose stimulation for 16 h can increase the expression of induced HIF-1α. Pretreatment with different concentrations of chrysin will inhibit the expression effects in a dose-dependent manner (Figure 6C).

### 2.4. Chrysin Down-Regulates VEGF Receptor Proteins and mRNA Expression

**Figure 7:**
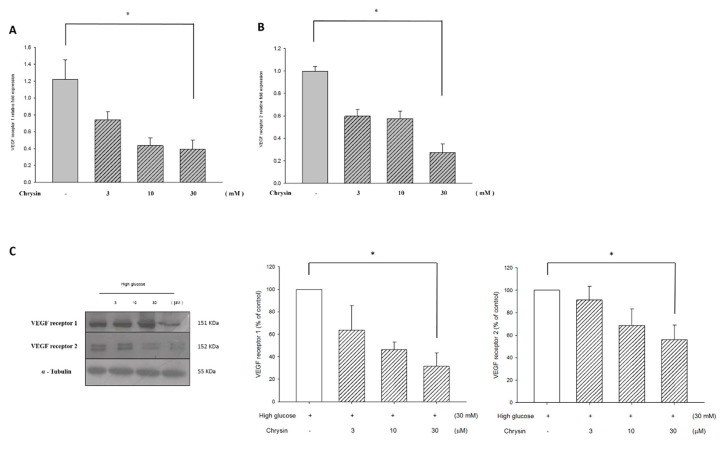
Chrysin decreased the mRNA levels of VEGF receptor1 (**A**) and VEGF receptor2. (**B**) RF/6A cells were treated with chrysin and then harvested after charging high glucose (30 mM) for 16 h, and qRT-PCR was performed for the mRNA expression levels. Results are expressed as a percentage of control and mean ± SE. *p < 0.05 vs. control. (**C**) Chrysin reduced the protein expression levels of VEGF receptors. RF/6A cells were treated with chrysin and then harvested after changing high glucose for 16 h. A western blotting was performed for the protein expression levels with the quantification data shown on the right panel. All data was acquired by means of ± SE from at least three independent experiments.

qRT-PCR was used to observe the mRNA expression of RF/6A cells after pretreatment of chrysin in a high glucose environment. Results showed that the mRNA expression of VEGFR decreased after pretreatment of chrysin (Figure 7A,B). Further observations of the relationship between the amount of the protein expressed and the mRNA expressed showed that the pretreatment of chrysin effectively down-regulated the protein expression of VEGFR (Figure 7C). These results indicate that chrysin might inhibit high-glucose induced chorioretinal endothelium migration by down-regulating the expression of VEGFR.

## 3. Discussion

DM basically means that the glucose present in blood cannot be used effectively. This leads to high glucose concentration and eventually, to glucose excretion in the urine. Hyperglycemia causes chronic inflammation and also promotes abundant cells to migrate, eventually moving to their final destination and beginning angiogenesis via factors including VEGF [16]. Many diabetic patients suffer from substantial vision loss due to proliferative retinopathy. Intravitreal injection of anti-VEGF drugs can have a positive effect on extensive retinal edema and proliferative vascular retraction. However, it cannot completely inhibit retinal angiogenesis for the reaction time requires less in vitro. Further, anti-VEGF drugs have also been found to have adverse reactions. At present, many people are increasingly interested in plant polyphenols as an alternative method to treat or prevent diabetic retinopathy [17,18].

In this study, chorioretinal EC (RF/6A) was used as the main cell model for retinopathy and cultured under high glucose conditions to mimic the environment of high blood glucose. Although the endothelial characteristics of RF/6A cells were questioned [19], abundant literature shows that RF/6A cells are still used as endothelial cell lines to model retinal and choroidal angiogenesis [20,21,22,23,24]. Culturing ECs under high-glucose conditions cannot mimic all characteristics of DM but might explain the endothelial growth factor autocrine mechanism of the ECs [25,26]. On the other hand, chrysin, a flavonoid found in propolis and honey, was chosen as the target agent. Its biological characteristics have been approved by previous studies. Our previous work, as well as other scholarly works, found that chrysin can provide antioxidant, anti-inflammatory, and anti-angiogenic effects [13,27,28,29,30,31,32,33,34]. The results of this study allow us to have better understanding of the possible pharmacological mechanism and biological activity of chrysin in DR. In the future, it can provide evidence for the prevention or treatment of retinopathy by utilizing chrysin.

Our results show that chrysin can inhibit high-glucose induced chorioretinal EC migration, even in the transwell assay or in the wound healing experiment with normal blood glucose condition (5.5 mM) as the control. We performed a survival experiment to make sure that the effect was not caused by cytotoxicity, but the decrease of cell migration. This experiment revealed chrysin had no cytotoxic effect on choridal ECs and did not influence chorioretinal ECs viabilities under high-glucose and high-osmotic conditions. According to the literature, mitogen-activated protein kinase (MAPK), and AKT played important roles in cell migration and cell survival [35,36,37,38]. The MAPK family includes members such as P38, ERK, and c-Jun N-terminal kinase (JNK). It has been proven that the MAPK family could regulate cell proliferation, cell migration, cell differentiation, and cell survival [39]. Moreover, in the development of tumors, ERK can regulate VEGF and HIF-1α activity [40,41]. On the other hand, AKT has also been proven to have a large amount of bioactivities, such as regulation of angiogenesis, cell migration, cell survival, and cell proliferation [42]. In one of our previous studies, lutein, a carotenoid, could down-regulate AKT phosphorylation in retinal pigmented epithelium (RPE) and thereby inhibit cell migration [36]. Thus, the pathway of the MAPK family and the role of AKT in chrysin’s regulation of cell migration were also investigated in the aforementioned study. From our results, it was speculated that chrysin inhibits high-glucose induced chorioretinal EC migration through down-regulation of AKT and ERK phosphorylation. AKT and ERK participated in the signal transduction of cell migration, but other members of MAPK included P38 and JNK showed no significant differences.

Extracellular matrix (ECM) consists of collagen, proteoglycans, carbohydrates, and glycoproteins. It acts as cell fixation, cell support, and regulation of signal transduction. Further, ECM has been proven to regulate tumor metastasis [43,44]. Matrix metalloproteinase (MMP) is a family of enzymes which can degrade ECM. The structure and function of most members of the MMP family have been widely understood. In particular, MMP-2 and MMP-9 are gelatinase and play important roles in cell migration and angiogenesis. They degrade gelatin and cause ECM to release signals related to cell migration or angiogenesis [44]. Our results confirm that chrysin can inhibit the expression of MMP-2 in a high-glucose environment, and this inhibitory phenomenon of MMP may be attributed to one of the mechanisms of chrysin’s inhibition of cell migration.

Previous studies have shown that VEGF expression increased in the serum and vitreous of patients has a positive relationship with blood glucose level [25,45,46]. In our study, we did find that the expression of VEGF in chorioretinal endothelial cells could be triggered in high glucose environment (Figure 6A). Then, the pretreatment of chrysin could inhibit the expression of VEGF (Figure 6B) and reduce the transcription factor HIF-1α (Figure 6C). Some previous studies have also shown that chrysin reduces the expression of HIF-1α by inhibiting the protein synthesis and stability of HIF-1α [8]. This, in turn, reduces the expression of VEGF and inhibits angiogenesis. Recently, chrysin was also found to inhibit laser-induced choroidal neovascularization (CNV) and down-regulate HIF-1α and VEGF expression [34]. Integrating these results, we speculate that the inhibitory effect of chrysin on the expression of HIF-1α and VEGF in a high glucose environment may contribute to the prevention or treatment of DR. This mechanism deserves further study.

Further, previous studies have also shown that chrysin inhibits LPS-induced angiogenesis by down-regulating VEGFR [14]. Consequently, we also investigated whether VEGFR could be related to the inhibitory effects of chrysin in a high-glucose related EC migration. Our results showed that expressions of VEGF receptor 1 and 2 proteins and mRNA were decreased under a high-glucose condition after pretreatment of chrysin. This effect might contribute to the inhibitory effect of chrysin and determine the expression of VEGF. We believe this is worth researching further. Moreover, we believe that the mechanisms underlying the patho-physiology and treatment of a disease are more complex and multi-factorial than in vitro cell experimental models. It is generally known that cultured cells do not entirely mimic diseased cells. However, the current data indicate that chrysin may have good potential in ocular neovascularization, such as choroidal neovascularization, diabetic retinopathy, etc. In the future, it is necessary to prove its efficacy through primary endothelial cells or in vivo experiments.

## 4. Materials and Methods

### 4.1. Cell Culture

The rhesus macaque choroid-retinal (chorioretinal) endothelial cell line RF/6A derived from the choroid-retina of a rhesus macaque fetus were purchased from Food Industry Research and Development Institute (Hsinchu, Taiwan). The cells were maintained with a low concentration of glucose (5.5 mM) Dulbecco’s modified Eagles medium (DMEM) as well as supplemented with 10% fetal bovine serum (FBS, Gibco, Gaithersburg, MD, USA) and 1% penicillin (Hyclone, UT, USA) at 37 °C, 5% CO_2_, and 95% humidified air. For most of the experiments, cells reaching 90–95% of confluence were synchronized for 24 h by serum starvation before they were subjected to further analysis.

### 4.2. Chrysin Treatment and High-Glucose Induction

Chrysin was purchased from Sigma-Aldrich, with a purity level of 97%. Chrysin was prepared by dilution with dimethyl sulfoxide (DMSO) to 30 mM and further to desired concentrations with culture mediums. RF/6A cells were cultured in a chrysin culture medium. The chrysin culture medium was removed after 24 h of treatment. A high glucose concentration of medium (30 mM) was added according to the demands of each experiment to induce damage and take mannitol (30 mM) as control group in hyperosmotic status.

### 4.3. MTT Viability Assay

The viability of the cells was decided by the MTT assay. Chrysin-treated RF/6A cells were incubated for 24 h. After a brief wash with the medium, 0.5 mg/mL MTT in DMSO was used for the quantification of living and metabolically active cells. Mitochondrial dehydrogenases in viable cells can reduce MTT to a purple formazan dye, which was analyzed by recording changes in absorbance at 550 nm using a spectrophotometer. Cell viability was proportional to the absorbance measured.

### 4.4. Transwell Assays

The transwell RF/6A cells migration assays were measured through a modified Boyden chamber model (Transwell apparatus, 8.0 µm pore size, Costar). For detection of RF/6A cell migration in the transwell, the lower faces of polycarbonate filters (Transwell insert) were coated with fibronectin (0.3 mg) 1 h before the experiment in the laminar flow hood. The lower chamber was filled with different concentrations of glucose. RF/6A cells (3 × 10^4^ cells, 200 μL) were plated to the upper chamber. After 5 h of incubation, the inserts were removed and the inner side was wiped with cotton swabs. All cells that had migrated were fixed and stained with 0.5% toluidine blue in 4% PFA. The migrated cells were counted as the number of stained cells per × 100 field (high power field, HPF) under a phase-contrast microscope (Leica DMIL1) and photographed.

### 4.5. Scratch Wound Assay

The culture plate with pre-treated RF/6A was scratched with a cross wound through the entire center of the well and then washed with PBS. The plate was photographed under a microscope and then filled with different concentrations of the glucose medium. After 24 h of incubation, the culture medium was removed and the cross wound was photographed under a microscope. The polygon selection mode was used to quantified the area of wound by Java’s Image J software (http://rsb.info.nih.gov). The percentage of wound closure was expressed: 

% of wound closure = [(A_t = 0 h_ − A_t = 24 h_)/A_t = 0 h_] × 100%

A_t = 0 h_: the area of wound measured immediately after scratching.

A_t = 24 h_: the area of wound measured 24 h after scratching.

### 4.6. Western Blotting Analysis

Western blotting analysis was used to understand the expression and activity of AKT (Cell Signaling: #4058, MA, USA), ERK (Santa Cruz: sc-7383, CA, USA), VEGF (R&D Systems: MAB293-100, MN, USA), HIF-1α (Cell Signaling: #14179, MA, USA), and MMP-2 (GeneTex: GTX104577, CA, USA), and all antibody dilution ratios were 1/1000. RF/6A cells pre-treated with different concentrations of chrysin were cultured with different concentrations of glucose. RF/6A cells were then lysed in a radioimmunoprecipitation assay buffer. After sonication, the lysate was centrifuged (13,200× *g* for 10 min at 4 °C) and the supernatant was removed. The protein content was quantified by a Pierce bicinchoninic acid (BCA) protein assay kit (Rockford, IL, USA). Total protein was separated by gel electrophoresis (10% SDS-polyacrylamide gels). The proteins were then electroblotted onto polyvinylidene fluoride (PVDF) membranes and probed using the indicated antibodies. Immunoblots were detected by enhanced chemiluminescence (Chemiluminescence Reagent Plus from NEN Life Science Products, Boston, MA).

### 4.7. Real-Time Quantitative RT-PCR

After chrysin pre-treatment and different concentrations of glucose medium culture, total RNA of RA/6A was isolated using the total RNA Plus Mini kit (Taigen Bioscience Corporation, Taiwan) according to the manufacturer’s instructions and reverse-transcribed into cDNA using the iScript^™^ cDNA Synthesis kit (Bio-Rad, Hercules, CA). The qPCR was performed using the StepOnePlus^™^ Real-Time PCR System (Applied Biosystems, Foster City, CA, US) with SYBR green (Applied Biosystems, Foster City, CA, US). Primer sequences used in the PCR reactions are VEGF, forward primer 5′-AGTTCCACCACCAAACATGC-3′, reverse primer 5′-TGAAGGGACACAACGACACA-3′; GAPDH, forward primer 5′-CTTTGGTATCGTGGAAGGACTC-3′, reverse primer 5′-GTAGAGGCAGGGATGTTCT-3′; VEGF receptor 1, forward primer 5′-GGGTCACATCACCTAACATCAC-3′, reverse primer 5′-CCTTTCTGCTGTCCCAGATTAC-3′; VEGF receptor 2, forward primer 5′-GACATGTACGGTCTACGCTATTC-3′, reverse primer 5′-CCTCCACACTTCTCCATTCTTC-3′. Data were normalized relative to GAPDH expression and evaluated using the equation: fold change = 2^−∆∆*C*t^.

### 4.8. Statistical Analysis

Results were analyzed with SigmaPlot for Windows (Version 10.00). The data was acquired by means of ± SE from at least three independent experiments. The *t*-test was performed to determine the difference between groups. A value of *p* < 0.05 was considered statistically significant.

## 5. Conclusions

In summary, previous published literature as well as our study suggest that chrysin has the bio-characteristics of inhibiting high-glucose induced cell migration. This conclusion helps the future development of nature product extraction. Currently, laser photocoagulation therapy is the main clinical treatment of diabetic retinopathy, even though it has the side effect of decreased visual sensitivity. This study provided important evidence for chrysin supplement as a good candidate in the treatment or even prevention of diabetic retinopathy.

## Abbreviations

VEGF	Vascular endothelial growth factor
VEGFR	Vascular endothelial growth factor receptor
DM	Diabetes mellitus
DR	Diabetic retinopathy
MTT	3-(4,5-Dimethylthiazol-2-yl)-2,5-diphenyltetrazolium bromide
HIF-1α	Hypoxia-inducible factor 1-alpha
MMP-2	Matrix metalloproteinase-2
qRT-PCR	Quantitative real time polymerase chain reaction
AKT	Protein kinase b
ERK	Extracellular signal-regulated kinase
ROS	Reactive oxygen species
LPS	Lipopolysaccharide
EC	Endothelial cells
MAPK	Mitogen-activated protein kinase
JNK	C-Jun N-terminal kinase
RPE	Retinal pigmented epithelium
ECM	Extracellular matrix
CNV	Choroidal neovascularization
DMEM	Dulbecco’s modified Eagles medium
FBS	Fetal bovine serum
DMSO	Dimethyl sulfoxide
PFA	Paraformaldehyde
HPF	High power field
PBS	Phosphate buffered saline
BCA	Bicinchoninic acid
SDS	Sodium dodecyl sulfate
PVDF	Polyvinylidene fluoride
SE	Standard error
